# The influence of tonality, tempo, and musical sophistication on the listener’s time-duration estimates

**DOI:** 10.1177/17470218231203459

**Published:** 2023-10-31

**Authors:** Ligia Borges Silva, Michelle Phillips, José Oliveira Martins

**Affiliations:** 1 Centre for Interdisciplinary Studies (CEIS20), Institute of Interdisciplinary Research, Faculty of Arts and Humanities, University of Coimbra, Coimbra, Portugal; 2 Royal Northern College of Music, Manchester, UK

**Keywords:** Musical tempo, duration estimation, time perception, tonality, musical sophistication, Gold-MSI, verbal estimation method, reproduction method

## Abstract

Music listening affects time perception, with previous studies suggesting that a variety of factors may influence this: musical, individual, and environmental. Two experiments investigated the effect of musical factors (tonality and musical tempo) and individual factors (a listener’s level of musical sophistication) on subjective estimates of duration. Participants estimated the duration of different versions of newly composed instrumental music stimuli under retrospective and prospective conditions. Stimuli varied in tempo (90–120 bpm) and tonality (tonal-atonal), in a 2 × 2 factorial design, while other musical parameters remained constant. Estimates were made using written estimates of minutes and seconds in Experiment 1, and the reproduction method in Experiment 2. Two-way analyses of variance (ANOVAs) showed no main effect of tonality on estimates and no significant interactions between tempo and tonality, under any condition. Musical tempo significantly affected estimates, with the faster tempo leading to longer estimates, but only in the prospective condition, and with the use of the reproduction method. Correlation matrices using the Pearson correlation coefficient found no correlation between musical sophistication scores (measured using the Goldsmiths Musical Sophistication Index [Gold-MSI]) and verbal or reproduction estimates. In conclusion, together with the existing literature, findings suggest that (1) changes in tonality, without further changes in rhythm, metre, or melodic contour, do not significantly affect estimates; (2) small changes in musical tempo influence only prospective reproduction estimates, with larger tempo differences or longer stimuli being needed to cause changes in retrospective estimates; (3) participants’ level of musical sophistication does not impact estimates of musical duration; and (4) empirical research on music listening and subjective time must consider potential method-dependent results.

Multiple factors influence our sense of time, and although this field of study has been active for over 100 years, there is still much we do not understand about the complexities of time perception. The recent “Listener Environment Music Interaction” (LEMI) model, which attempts to draw together the findings of existing experiments on how music listening impacts on sense of time (or duration), lists three main relevant factors: the listener (including aspects such as age and previous musical experiences), the musical features (characteristics of the musical stimulus), and the context (the mediating condition through which music is heard, including social context, venue, environmental conditions, etc.) (Phillips, 2022). Perception of duration also varies according to whether the individual is aware of time passing and actively tries to estimate the duration of ongoing events (prospective timing) or attempts to estimate the duration of past events after they have taken place (retrospective timing).

The present experiments focus on two factors of the LEMI model: musical features, namely, the effects of the presence/absence of a pitch hierarchy and changes in musical tempos, and the listener, namely, their degree of musical sophistication. The literature which follows, outlines previous experimental work related to tonality, musical tempo, and musical sophistication, and their influences on time perception.

## Pitch hierarchies

Although pitch can be heard as a continuously scaled percept, musical traditions across most cultures use only a set of discrete pitches, which often follow a hierarchy of stability and relevance, for example, a major, minor, or pentatonic scale (Bigand & Poulin-Charronnat, 2016; Stainsby & Cross, 2016). For the majority of listeners, stable pitches have a conclusive and relaxed feeling, while unstable pitches tend to be felt as inconclusive and create tension (Bigand & Poulin-Charronnat, 2016; Koelsch, 2013). The alternation between less and more stable pitches (or chords) may evoke a sense of tension and release. After sufficient exposure to these patterns in tonal music, listeners’ expectations revolve around the anticipation between tense and later relaxed moments (Huron, 2006). This, in turn, can lead to the formation of melodic and harmonic expectations (Krumhansl & Cuddy, 2010), where experienced listeners can anticipate which exact pitch will be the more stable one, following a chord or a scale (Krumhansl & Kessler, 1982; Krumhansl & Shepard, 1979; Sears et al., 2018). Once they are internalised, pitch hierarchies (such as the tonal hierarchy) become stable and abstract constructs, which include information about the intervallic disposition of a pitch set and the different degrees of stability that arise from it. According to Fred Lerdahl (2001), “Such a hierarchy is atemporal in that it represents more or less permanent knowledge about the system rather than a response to a specific sequence of events” (p. 41). Although a tonal hierarchy is an abstract and static structure stored in long-term memory (Bigand & Poulin-Charronnat, 2016), the tonic (the reference pitch) is defined within each musical piece and has to be kept in working memory. Studies have suggested that tonal hierarchies may be acquired during childhood through acculturation, without formal training (Krumhansl & Cuddy, 2010; Matsunaga et al., 2019). Empirical work has shown that adult listeners unfamiliar with a given pitch hierarchy can learn it after a short exposure period during an experiment (Krumhansl, 2000; Loui et al., 2010; Stevens et al., 2013).

The concept of a tonal hierarchy stipulates how pitches in a scale relate to one another, a specific example of which underlies a significant amount of music (so-called “tonal music”) heard in Western cultures today, across multiple music genres. It is worth noting, however, that non-Western cultures use a variety of differently constructed pitch hierarchies. In addition, significant stylistic strands in Western music are *not* regulated by tonal hierarchies. For example, in the early 20th century, composers explored a variety of styles and pitch combinations (usually labelled as atonal and serial music), which denied pitch hierarchies, and where all pitches were often compositionally designed to have the same level of importance.

The perception of tonal and atonal music has been examined in a number of studies. Some of those studies discuss the reasons for possible relationships between the sense of tonality produced by a piece of music and how it is perceived in terms of duration. These studies have forefronted the concepts of familiarity and pleasantness, which will now be discussed.

### Tonal and atonal music—effects of familiarity and pleasantness on attention and duration estimates

According to the literature on time perception, the perceived duration of an event is associated with the amount of attention one dedicates to it, and to which features of the event it is directed (Grondin, 2010; Wearden, 2016). In a retrospective situation, following memory-based models, duration may be estimated according to the amount of information processed and memorised from an event (Ornstein, 1969). In this case, paying greater attention to an event will cause it to be perceived as longer, as more information is memorised and later used to estimate duration. In a prospective situation, following internal clock models of time perception, duration may be estimated according to the number of stored inner pulses, which work as time units (Gibbon et al., 1984; Zakay & Block, 1994). In this case, paying greater attention to the non-temporal features of an event causes it to be perceived as shorter, as less attention is directed to tracking time, which leads to some inner pulses not being stored.

In music listening, the amount of attention dedicated to the music seems to be influenced by the perceived degree of familiarity with the musical content, and the music-induced pleasantness. In a study about music’s familiarity and subjective duration, Bailey and Areni (2006) found that familiar music of about 3 minutes attracted listeners’ attention more than unfamiliar music, leading to shorter estimates in a prospective paradigm, and longer estimates in a retrospective paradigm. To most Western listeners, tonal music will tend to sound more familiar than atonal music, as evidenced in an experiment using tonal and atonal versions of a selection of Beethoven’s Sonatas (Lalitte et al., 2009), perhaps because tonality is more present in their respective musical environments than atonality. Considering this, it is expected that studies investigating the effects of tonal and atonal music on duration estimates will have similar results as those obtained by Bailey and Areni (2006) about music’s familiarity and time perception, with listeners’ attention focusing more on tonal music than atonal music. This would lead to tonal music being perceived as longer retrospectively and shorter prospectively, when compared with atonal music. Such results were found in an experiment conducted by Ziv and Omer (2010), where Bach’s music was rated as shorter than Schoenberg’s music in a prospective paradigm, but longer than it in a retrospective paradigm. These findings suggest that Bach’s music attracted more listeners’ attention than Schoenberg’s music, which can be attributed to a higher familiarity with the musical features present in Bach’s music (tonality being one of them). However, in Ziv and Omer’s experiment, it is hard to determine whether participants had different degrees of familiarity with Bach’s or Schoenberg’s music in general, or with any specific feature of these compositions. So, in the study reported here, we assumed the method of using newly composed musical sentences to ensure that participants had no previous familiarity with the stimuli, and that changes in familiarity may only be attributed to changes in the use of pitch hierarchies. Three other studies found that tonal music tended to be estimated as shorter than atonal music in a prospective approach, or as longer than atonal music in a retrospective approach (Droit-Volet et al., 2010; Droit-Volet et al., 2013; Kellaris & Kent, 1992).

More generally in the field of time perception, in experiments not using music as stimuli, familiarity seems to impact time perception differently. Block et al. (2010) analysed familiarity as one of the factors that change the cognitive load associated with a task, with lower familiarity being associated with a higher cognitive load. In this meta-analysis the authors summarised that an increase in the cognitive load associated with a task would create a higher demand for attention. This would lead to shorter estimates in a prospective approach (where, according to an internal clock perspective, an increase in the cognitive load of a task would lead to less attention being dedicated to tracking time, shortening the number of pulses stored and the resulting duration estimate) and longer estimates in a retrospective approach (where, according to a memory-based perspective, an increase in the cognitive load of a task would lead to a higher amount of information processing, resulting in longer estimates of duration). The same reasoning is the basis of the attentional gate model proposed by Zakay and Block (1994), which assumes that the perception of unfamiliar stimuli requires higher cognitive engagement. However, Block et al. (2010) concluded that familiarity affected only retrospective and not prospective duration judgements, suggesting that it may affect memory encoding and retrieval, but not attentional resource allocation. Considering this conclusion, one would expect that atonal music would attract greater attention than tonal music, given that it is less familiar, especially in a retrospective setting. On the contrary, empirical evidence on tonal and atonal music has suggested that not only in music listening a lower level of familiarity seems to be associated with less attention paid, but this association also seems to occur in both prospective and retrospective situations. The evidence then suggests that the relationship between familiarity and attention may work differently in relation to music listening than in relation to other tasks which have been explored experimentally. Jones (2014) already noted that the particular case of music listening may require a new conception of psychological time, given that due to the inherent temporal dimension of the musical expression, time is difficult to dissociate from the structure of the musical event itself. In addition, it has been proposed that how perceived familiarity affects the willingness to pay attention to music is dependent on the listeners’ musical preferences and personalities, such as openness to novelty (Mencke et al., 2019). This possibility may be connected with the degree of enjoyment that music can provide, and how enjoyment may relate to perceived familiarity.

Not only does tonal music tend to be more familiar to most listeners when compared with atonal music, but it is also often judged as more pleasant and as linked with more positive valence (Droit-Volet et al., 2010, 2013; Kellaris & Kent, 1992; Mencke et al., 2019; Ziv & Omer, 2010). Block et al. (2018) point to the common saying that *time flies when you’re having fun*, to explain that a task demands a certain level of attention according to its perceived pleasantness, and not to its complexity. Kellaris and Kent (1992) refer to both familiarity and musical preference: “There may be a tendency to devote more attention to liked music or to retain more information from familiar types of music, both of which may influence perceived duration” (p. 368). A connection between music-induced enjoyment and perceived duration was reported in two studies (Droit-Volet et al., 2013; Phillips & Cross, 2011). While in Droit-Volet et al.’s (2013) study, which used a prospective setting, ratings of higher pleasantness attributed to the music resulted in shorter estimates, in Phillips and Cross’s (2011) retrospective study, listeners who reported more enjoyment during music listening also judged the musical excerpts as longer than listeners who found music less enjoyable. Together, these studies suggest that the more enjoyable the music is perceived to be, the more it will attract the listener’s attention, resulting in more information being stored in memory.

It is also possible that tonal and atonal music lead to different duration estimates due to their different potentials to give rise to musical expectations. Musical expectation is one of the emotion-inducing mechanisms activated during music listening, as described by Juslin (2019). As described earlier, tonal music is more likely to induce expectations of occurrence of specific pitches and tonal resolution than atonal music. Given that some studies suggest that emotions may affect subjective time (Wearden, 2016), a possible interaction between duration estimation processes and emotion-inducing mechanisms motivated by musical expectations may result in different estimates being attributed to tonal and atonal music.

Taken together, the literature described above suggests that, due to its higher perceived pleasantness and familiarity, tonal music will tend to be judged as shorter than atonal music, prospectively, and as longer than it, retrospectively.

## Musical tempo

Any piece of music can be played faster or slower. That speed is usually referred to as musical tempo. In most musical pieces, sounds can be perceived as being organised in a regular temporal grid. Those pieces are often described as having a pulse, or a beat. The ability to extract (or “infer”) a regular pulse from an auditory stimulus is what allows humans to synchronise movements with music, such as dancing or clapping (Kozak, 2020; Trainor & Corrigall, 2010). Composers may state the musical tempo of their compositions by specifying the number of beats per minute (bpm) and identifying which rhythmic figure should be taken as the beat (this instruction is called a metronome mark). Music often has a regular metre, meaning that beats can be hierarchically organised with a downbeat (a more relevant, reference beat) happening regularly, most commonly, every two, three, or four beats. A beat can also be subdivided into two or three shorter durations. This creates several temporal levels, organised hierarchically (labelled metric levels) that can be shorter, equal, or longer than the beat (Lerdahl & Jackendoff, 1983; London, 2004). Any regular pulse may then evoke the feeling of secondary pulses with rate multiples of its own, evidenced by the occurrence of multiple and synchronised active neural oscillations, described by Jones (2019) as metric clusters. For this reason, there may be a difference between the metronome mark of a given musical piece and the metrical level that a listener feels as the most salient pulse (London, 2011). While hearing a piece with the metronome mark of 200 quarter-notes/min, a listener may feel the half-note to be the most salient pulse (a half-note equals two quarter-notes, representing one metrical level higher), which would equal a 100-bpm tempo from the listener’s perspective. Two pieces with the same metronomic mark may also be subjectively felt as faster or slower depending on their rhythmic density (the number of musical events sounding per time unit) (London, 2011). For instance, a musical excerpt with events sounding only every two beats may be felt as slower than its metronomic mark, as the listener may assume each musical event to be one beat. Other musical features such as loudness, timbre, and register also seem to affect the perceived musical tempo (Boltz, 2011). Perception of musical tempo is then a subjective phenomenon, affected by the speed at which the music is played, other musical features, and personal factors that determine how each listener perceives those features and organises the temporal information conveyed in the music.

### Spontaneous motor tempo and preferred perceptual tempo. The perception of musical tempo

Spontaneous motor tempo (SMT) is the rate at which one executes spontaneous and regular movements, such as walking or clapping, without focusing consciously on their speed. It is generally explored experimentally by asking participants to tap a regular beat at their most comfortable speed. Across different studies, the most representative value for SMT in adults is 600 ms (McAuley, 2010). Although SMT can vary widely between different individuals (ranging between 300 and 800 ms), it tends to be fairly stable within a given production, varying only by 5% (McAuley, 2010). SMT is not fixed and can vary according to age (slowing down as we get older), arousal (faster in higher arousal states), and circadian rhythms (usually faster during the day and slower during the night) (Hammerschmidt & Wöllner, 2023; McAuley, 2010). Preferred perceptual tempo (PPT) is another measure used in studies on the perception of musical tempo. PPT refers to the temporal rate of regularly occurring events that feels “just right”: neither too fast nor too slow. While SMT involves motor activities performed by the participant, PPT involves the observation and perception of regularities that may be presented in the form of movements, sounds, or lights. PPT tends to assume values close to the ones observed for SMT (McAuley, 2010). The connection between SMT and age makes it predictable that listeners of different ages may prefer music at different tempos.

A study conducted by McKinney and Moelants (2006) found that the perception of a musical pulse varies across individuals, depending on the characteristics of the musical content. In this experiment, listeners were instructed to tap to the pulse they felt was “the most salient,” while listening to several different pieces of music at different tempos and from different genres. Although for the majority of the stimuli there was one metrical level clearly defined as the preferred by most listeners, in some cases, up to four different pulses were tapped by different listeners, for the same piece of music. Also, the musical genre had a significant effect on the variety of metrical levels chosen and on the number of participants tapping to the most salient tempo. Individual differences in tapping preferences were also evidenced, with studies finding that some participants consistently tap either to the slower or the faster metrical level available, across musical examples in different tempos (London, 2011; McKinney & Moelants, 2006). The reasons behind these particular individual choices are still unclear. McKinney and Moelants (2006) point to musicianship, age, and culture as possibly relevant factors. In a similar study using six different rhythmical patterns as stimuli, Parncutt (1994) found that when rhythmic patterns are played slowly, tappings tended to match the quarter-note, which was considered as the beat in this study. As speed increased, the rate at which people tapped coincided more with the longer notes of the pattern and with the music-theoretical downbeat. In summary, the perception of musical tempo is a subjective phenomenon not yet clearly understood, with empirical evidence suggesting that it may depend on musical factors (such as musical genre and the speed at which music is performed) and individual factors (such as age, culture, or musical training).

### Studies on the impact of musical tempo on time perception

As Phillips (2022) recently summarised, studies about the influence of musical tempo on duration estimates often have inconclusive or contradicting findings. While some experiments found that changes in tempo affected duration judgements (Biasutti & Pattaro, 2006; Droit-Volet et al., 2013; Hammerschmidt & Wöllner, 2020; Mailov, 2011; Oakes, 2003), others did not observe such effects (Caldwell & Hibbert, 2002; Cassidy & Macdonald, 2010; North et al., 1998).

Studies that found a relationship between musical tempo and duration estimates often reported that a faster tempo led to longer estimates. This was found to be the case in prospective studies (Droit-Volet et al., 2013; Hammerschmidt & Wöllner, 2020; Hammerschmidt et al., 2021), and in studies described as retrospective (Mailov, 2011; Oakes, 2003). In studies where a faster tempo led to longer estimates, it has often been hypothesised that a faster tempo corresponds to a higher amount of information being processed when compared with a slower tempo (Phillips, 2022). This would explain retrospective estimates, since, according to memory models of time perception, higher amounts of information processing or cognitive effort are associated with longer estimates. In the case of prospective studies, one can hypothesise that a faster musical tempo can accelerate the internal clock either through arousal or entrainment (i.e., the listener inferring a faster pulse than with a slower piece of music), which would result in more accumulated pulses and longer estimates. The arousal link was proposed by Droit-Volet et al. (2013) when discussing their findings in a prospective study about music, emotion, and time perception. Juslin (2019) proposes rhythmic entrainment as an emotion-inducing mechanism, suggesting that the locking of an internal rhythm (such as heart rate) to a faster musical tempo may lead to a more aroused state. Evidence suggests that entrainment may also happen with simpler and non-musical stimuli. Listening to faster temporal cues, such as simple sound clicks, seems to be linked to longer estimates of duration. This has been found when participants were solely occupied with hearing those clicks, both in retrospective and prospective settings (Polkosky & Lewis, 2002; Wearden, 2016) and when cues occurred in the background while participants performed other tasks (Zakay et al., 1983). This suggests that recurring auditory events may affect temporal judgements even when listeners are not directly focused on them, a possibility that relates to the limits of introspection referred to by Juslin (2019), who notes that emotions can be elicited by inputs which one is consciously unaware of.

In a study focused on a more active music listening experience, Hammerschmidt and Wöllner (2020) found that either tapping to different metrical levels during music listening or simply focusing attention on those levels tends to lead to different duration judgements. In this study, when participants tapped or focused on longer rhythmic figures, such as half-notes, they judged the musical examples as shorter than when they tapped or focused on shorter figures, such as eight-notes. The authors highlight the need for further research on music listening and duration estimation, which considers pulse salience and uses more realistic musical examples. It is important to note that both tapping to the beat or just focusing on it seems to produce the same results, either in terms of comparing the tempo of musical excerpts (London, 2011) or estimating musical duration (Hammerschmidt & Wöllner, 2020).

The experiments mentioned in this section differed widely in context (attentively listening to music vs music in the background, as a secondary task), and in the musical stimuli used, both in terms of duration (from a few seconds to several minutes) and in ecological validity (from sets of music of different genres to non-melodic rhythmic patterns or simple clicks).

In short, even though there is no consensus between all literature, there is a considerable amount of evidence suggesting that, across different contexts, a faster musical tempo may lead to longer estimates when compared with a slower tempo, either prospectively (through an increase in arousal or direct entrainment of an internal clock mechanism to a faster pulse) or retrospectively (through an increase in the information processed and cognitive load).

According to this, the second hypothesis to be tested in this experiment is whether a faster tempo leads to longer prospective and retrospective estimates.

## Pitch and time interaction

Although pitch and duration may be defined as two independent sound properties, they are perceived simultaneously and a possible interaction between them cannot be excluded. An inseparable nature of pitch and time dimensions in music perception was suggested by a series of experiments performed by Prince (2011), where changes in pitch affected the rating of how metric a melody was, and changes in rhythm affected the perception of tonality. It is possible, then, that this indissociable nature also reflects some interaction on how these dimensions affect duration estimates.

## Musicians and non-musicians

Through hours of deliberate practice (practice with clearly defined goals, a feedback mechanism, and constant pushing out of one’s comfort zone), musicians develop the ability to play an instrument and form highly detailed and meaningful mental representations of music (Ericsson & Pool, 2016). These mental representations allow them to process more musical material, and process this at a faster rate than non-musicians, and to think about possible transformations and new information, imagining changes and new musical content. According to Ericsson and Pool (2016), the quality and quantity of mental representations, and the ability to create new material and work with them in a certain domain is what defines an expert. However, besides deliberate practice, some music listening skills can still be acquired through exposure and engagement with music, causing “non-musician listeners” to have different abilities to process music, which can be considered as different degrees of musical expertise (Müllensiefen et al., 2014).

Evidence is still unclear regarding whether musicians and non-musicians differ in how they perceive music and how they judge the duration of musical pieces. Some studies suggest that the perception of tonality is influenced by the listeners’ level of musical training (Krumhansl & Shepard, 1979; Ockelford & Sergeant, 2012). This may cause changes in the use of pitch hierarchies to become more noticeable to some listeners, who may then be more subject to alterations in their temporal experience caused by those changes. On the contrary, as described previously, tonal hierarchies are acquired through acculturation, and unfamiliar pitch hierarchies and musical grammars seem to be learned passively and relatively fast. Rohrmeier et al. (2011) found that both musicians and non-musicians were sensitive to the presence of a new underlying grammar of melodic structure. Musicians and non-musicians seem to process tonal and metric hierarchies equally well (Prince, 2011). Correia et al. (2023) observed that musically untrained adults could detect melodic and rhythmic similarities as well as (or better than) adults with more than 6 years of musical training. Other musical perception processes, such as music structuring and segmentation, also seem to be independent of musical training, as shown in an experiment using contemporary music (Phillips et al., 2020). It must also be considered, however, that although in some of the above-described studies participants seem to process music similarly, that particular task may have represented different degrees of cognitive effort for participants with different musical training, which could impact duration judgements. Supporting this possibility is the finding from a retrospective experiment, which found that musicians judged the duration of an excerpt of a solo piano piece of music as significantly shorter than non-musicians (Phillips & Cross, 2011).

Contrary to tonal music, atonal music may be familiar to a smaller group of listeners as this kind of music is less commonly listened to worldwide (Mencke et al., 2019). Melodic expectations for atonal music seem to be affected by familiarity. In an experiment using the probe tone method, listeners more familiar with atonal (or in this case “serial” music, which is a form of atonal music in which all 12 chromatic notes are placed in a row without repetition, and this forms the theme, or melody) were able to learn the logic of non-repetition of the series: after hearing an incomplete series they rated the notes not yet heard as better endings than the ones already heard (Krumhansl et al., 1987). Participants unfamiliar with atonal music did the opposite, rating the notes heard more recently as better endings. The doubt remains regarding whether the perception of atonal music is facilitated by higher musical training, higher levels of exposure, or a combination of both, as noted by Phillips et al. (2020). Considering all previous research, one may expect that listeners with more musical training and a greater degree of familiarity with atonal music may feel fewer differences in the attention and cognitive effort required to process tonal and atonal music, which may lead to smaller differences in judgements of the duration of these two kinds of music. Conversely, a listener only familiar with tonal music may find that a higher degree of effort is needed to process the unfamiliar logic of atonal music, which may result in more distinct duration estimates between those two types of pitch organisation.

Musicians and non-musicians also differ in their knowledge of the bodily experience of playing an instrument. As Kozak (2020) states, when we listen to a recording we mentally construct the idea of a body that produced those sounds. Musicians and people with a greater level of musical training may be more familiar with live music and how instruments are played, which may cause them to better imagine the objects and bodily movements involved in the performance of the music they are listening to. Phillips (2014) notes that our experience of an event includes the temporal experience of that event. Our concept of 5 s is inseparable from our temporal experience of living a 5-s interval, including, for instance, memories of events that one knew lasted for that interval. Connecting this point of view with Kozak’s preposition, one may imagine that the experience of listening to music includes not only the temporal experience of that listening moment but also the experience, or even imagination, of the bodily movements one imagines associated with that sound production, which are also associated with their own temporal experience. This connection between body, music, and time perception suggests that musicians may perceive musical time differently than non-musicians, as they have personal knowledge that comes from the particular bodily experience of producing music and the inherent temporal experience that comes from enacting those movements. A question arises about whether this particular kind of bodily and temporal knowledge is reflected in the individual’s judgements about the duration of a musical piece and the perceived passage of time.

Experimentally, musical expertise has been assessed mainly through inquiries about musical training (hours of instrumental practice or years of formal music education) and aural tests. Müllensiefen et al. (2014) undertook a series of studies on assessing “musical sophistication.” This term (used by the authors and adopted here from now on) aims to describe the multifaceted nature of musical expertise, considering listeners’ abilities and behaviours that are not usually considered when determining musical expertise experimentally (Zhang et al., 2020). These authors created a self-assessment questionnaire: the Goldsmiths Musical Sophistication Index (Gold-MSI). Gold-MSI scores significantly correlated with scores from three different aural tests (Gordon’s AMMA, a melodic memory test, and a beat perception test), supporting the authors’ hypothesis that this questionnaire may be a valuable tool to assess musical sophistication. The questionnaire comprised five subscales: *Active engagement, Emotions, Perceived abilities, Musical training, and Singing abilities*. The three latter subscales are more relevant to this study, as they were shown to correlate more highly with scores from aural tests, and they focus on hours of deliberate practice, and the ability to form and create mental representations of music, both important factors in Ericsson and Pool’s (2016) concept of expertise. Since musical sophistication affects how music is mentally represented, one may expect that it also influences how listeners process the temporal information contained in the music. Hence, the third hypothesis addressed in this study is that the participants’ level of musical sophistication and/or the ability to play a musical instrument will affect duration estimates. To test this hypothesis, participants’ musical sophistication was assessed using a reduced version of the Gold-MSI, in both experiments described and documented below.

In summary, these factors—tonality/atonality, musical tempo, and the level of musical sophistication—have been shown to affect the temporal experience associated with music listening. Tonal music has been associated with longer retrospective estimates and shorter prospective estimates when compared with atonal music. Findings on the effects of musical tempo on duration estimates are less consensual, with some studies suggesting that a faster tempo may lead to longer estimates, both prospectively and retrospectively, while others report no effects of tempo on duration judgements. The potential influence of musical sophistication on estimates is also not clearly defined yet, with studies presenting contradictory results. The studies reviewed above often differ regarding the environment where music listening occurs (lab experiment vs. real-life situation), the contextual role of music (music listening as the central task vs. music in the background), and the complexity and ecological validity of the musical stimuli used.

The experiments reported here were conducted online, with music listening as the main task, and employing both retrospective and prospective paradigms. The stimuli differ from previous studies by effectively isolating both musical tempo and pitch hierarchies while keeping the musical stimuli ecologically valid (recorded with acoustic instruments, and including rhythmic, metric, and melodic parameters). To reduce experiment-induced familiarity distinct versions of two different melodies were used, and randomised, to avoid recognition of similarities and memorisation. Since we wanted to prevent the occurrence of veridical expectations (described by Huron (2016) as the kind of expectations listeners experience when they are already familiar with the particular piece of music they are listening to), musical stimuli were selected from a set of melodies newly composed for a previous experiment. These experiments tested three main hypotheses given below.

### Tonality

Tonal music will be judged as longer retrospectively, and shorter prospectively, when compared with atonal music.

### Tempo

A faster musical tempo will lead to longer duration estimates, in both prospective and retrospective paradigms.

### Musical sophistication

Musical sophistication and/or the ability to play an instrument will influence estimates of musical duration.

## Experiment 1

### Method

#### Participants

A total of 150 participants were recruited. All participants engaged in the retrospective condition and 95 of those engaged also in the prospective condition. The sample size was determined with an a priori power analysis using G*Power taking into account the analyses planned: a two-way analysis of variance (ANOVA) to study the effects (and possible interactions) of tempo and tonality on estimates, and a correlation analysis to study possible relationships between estimates and individual variables, such as Gold-MSI scores or amounts of attention. This was based on a moderate effect size (*f* = 0.25), an alpha level of .05 and a power of .8, and resulted in a calculation of 128 participants being required. Since in the prospective condition participants listened to several stimuli, providing four observation points, the number of recruited participants was higher than the required sample size, both in prospective and retrospective conditions. Participants were recruited through social media, the Prolific Academic platform (where people sign up to be alerted when surveys become available and are paid by researchers for their time completing these), and the SurveyCircle platform (where researchers engage in each others’ surveys voluntarily [only 55 of the participants used this platform, and all took part in the retrospective condition]). Musical expertise was calculated using the Gold-MSI scoring app, as mean scores for each subscale, ranging from 1 to 7 points. Table 1 describes the age and Gold-MSI subscales scores of participants engaging in the prospective and retrospective conditions.

**Table 1.** Descriptive statistics of age and musical expertise in retrospective and prospective conditions, in Experiment 1.

	Retrospective		Prospective	
	*M*	*SD*	*M*	*SD*
Age	33.71	11.68	37.81	12.23
Active engagement	4.65	1.48	4.68	1.58
Perceived abilities	4.91	1.14	4.91	1.22
Musical training	2.74	1.54	2.99	1.52
Singing abilities	3.81	1.50	3.92	1.59
Emotion	5.83	1.02	5.72	1.09
General sophistication	3.79	1.14	3.91	1.24

*SD*: standard deviation.

The study was granted ethical approval by the Centre for Interdisciplinary Research, based at the University of Coimbra, Portugal.

#### Materials

The study used eight different musical stimuli previously tested in a pilot experiment, which examined the perception of tonality and musical tempo (Silva et al., in preparation).

The stimuli used were systematically varied in tempo and tonality. The atonal versions were based on the tonal versions and were altered by shifting the pitch of each note by either a half (semitone) or a whole step (tone), higher or lower. In this transformation, the rhythmic and metric structures were preserved, as well as the melodic contours. The atonal versions avoided melodic movements that could create perfect triads, which could be perceived as resembling excerpts of major or minor scales containing the tonic, or containing intervals strongly associated with tonality (such as ascending perfect fourths or descending perfect fifths). Pitch repetition was avoided among the first 10 tones of the atonal versions, to simulate a serial melody, and make the perception of pitch hierarchies by participants even less likely.

The pilot test included four pairs of melodies (each pair included tonal and atonal versions of the same melody), in three different musical tempos (90, 120, and 150 bpm), and used the probe tone method (Krumhansl & Shepard, 1979) to test the perception of pitch hierarchies. Based on this test, the two pairs of melodies considered by participants to best represent distinctive tonal and atonal versions of the same original melody were selected to be used in this experiment. In the pilot test, participants also tapped while they listened to each melody (and submitted this tapping as a sound file to the experimenters), in order for the metrical level that they entrained to be clear. This was important since the literature has suggested that the perception of the most salient pulse can vary among individuals (see introduction above). The test revealed that listeners tended to spontaneously tap to lower metrical levels when stimuli were presented at 90 and 120 bpm, but shifted to higher levels once stimuli were presented faster, at 150 bpm. In this case, listeners’ perception of the salient pulse as a hypothetical quarter-note in a 90-bpm excerpt, and as a hypothetical half-note when the same excerpt was played at 150 bpm (a 75-bpm pulse) means that listeners assumed a slower pulse when the excerpt was, in fact, played faster, as they entrained to a higher metrical level. As one of the aims of this experiment was to test the hypothesis of entrainment of the internal clock with the perceived musical tempo, it was important to use two different musical tempos in which listeners still assumed the same metrical level as the salient pulse. Therefore, we decided to use distinct but relatively close tempos: 90 and 120 bpm. Considering all the insights gathered in the pilot test, we expect that in this experiment participants will perceive the atonal and tonal versions accordingly, and entrain to the 90 and 120 bpm periodicities as the most salient pulses in the presented stimuli. The eight different stimuli used in this experiment are summarised in Tables 2 and 3.

**Table 2.** Four stimuli originated by Melody A.

Tempo	Tonal	Atonal
90 bpm	Melody A—Tonal at 90 bpm	Melody A—Atonal at 90 bpm
120 bpm	Melody A—Tonal at 120 bpm	Melody A—Atonal at 120 bpm

**Table 3.** Four stimuli originated by Melody B.

Tempo	Tonal	Atonal
90 bpm	Melody B—Tonal at 90 bpm	Melody B—Atonal at 90 bpm
120 bpm	Melody B—Tonal at 120 bpm	Melody B—Atonal at 120 bpm

Melodies A and B differed in length (both in terms of the number of measures and seconds), melodic contour, and rhythmic organisation. The musical stimuli’s duration ranged from 16 to 26 s. Stimuli were played on the flute by a professional flautist and recorded using a Rode N1 microphone and a Solid State Logic interface. The audio recordings used in the experiment can be found in https://figshare.com/s/09755cd65c71f9d3dd2f, and scores are included in the Appendix.

#### Procedure

**Figure 1.**
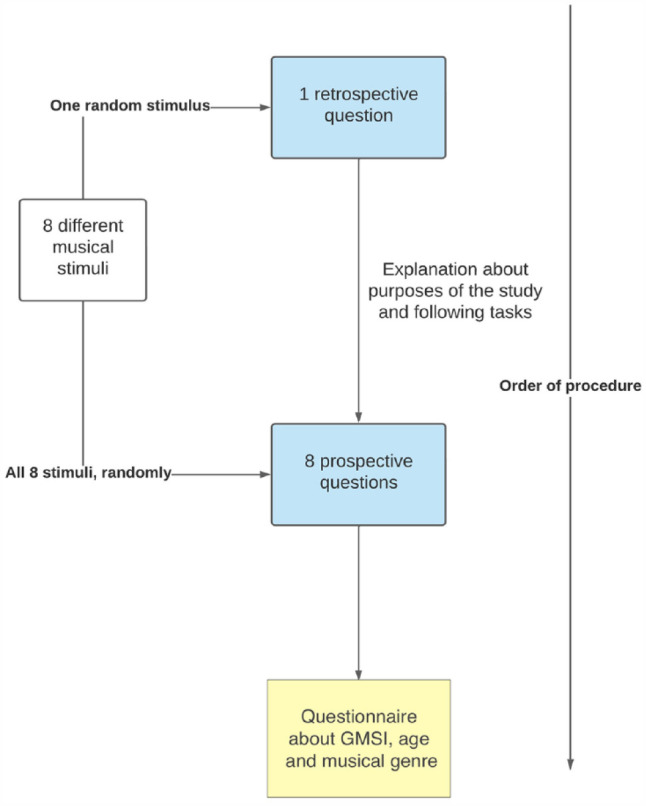
Experimental procedure.

The experiment was conducted through an online survey created and hosted on Limesurvey. Limesurvey allows customisation of surveys through HTML and Javascript coding, stores and fully anonymises responses, and hosts surveys in a web link. The survey link was then provided to participants recruited via social media, SurveyCircle, and Prolific Academic. An initial page informed participants that their answers would be anonymised and that by proceeding they were thereby consenting to their responses being analysed and published in articles reporting this experiment. The initial instructions asked participants to listen to the music with headphones, in a quiet environment free of distractions, and to only proceed if those conditions were met. After listening to the first stimulus (randomly selected out of the eight possible stimuli), participants were asked to estimate its duration without looking at a clock, by filling in blank spaces corresponding to X min and X s. This first estimate was hence a retrospective one, as participants did not know they would be making an estimate of duration until they had listened to the stimulus. After a brief explanation about why it was not initially mentioned that the experiment focused on time perception, participants were informed that they would repeat this task a few more times and that they should avoid counting or looking at clocks. Participants repeated the task of listening and immediately estimating the duration of each stimulus (in writing, expressed in X min and X s) for all the eight stimuli, in a randomised order. The stimulus heard first, in the retrospective condition, was included again in the prospective condition. After each estimate, participants were asked to rate how much attention they felt they had given to the stimulus, using a 5-point scale from (1) *very distracted* to (5) *very focused on the music*. At the end of the survey participants answered a reduced version of the Gold-MSI containing 19 questions (3 for each subscale plus 4 extra questions about instrument playing and absolute pitch) questions about demographic data (age, country of birth, and country of residence) and musical preferences according to musical genres. The complete procedure is summarised in Figure 1.

#### Data analysis

First, participants’ estimates of duration were divided by the real chronometrical duration of each stimulus. The result, from now on referred to as the “relative estimate,” represents the proportion between estimates and real duration, with values >1 indicating an overestimation, and <1 indicating an underestimation. Second, outliers were excluded using the Jamovi software feature for labelling outliers, which used the following rules:

The upper whisker extends from the hinge to the largest value no further than 1.5 * IQR from the hinge (where IQR is the inter-quartile range, or distance between the first and third quartiles). The lower whisker extends from the hinge to the smallest value at most 1.5 * IQR of the hinge. Data beyond the end of the whiskers are called “outlying” points and are plotted individually.

Third, for each participant, we calculated the averages of estimates for stimuli with the same conditions (e.g., estimates for Melody A Tonal at 90 bpm and Melody B Tonal at 90 bpm resulted in an average for the “Tonal + 90 bpm” condition). These averages (referred to as the “prospective relative estimates”) were used when analysing the possible effects of the musical variables on prospective estimates. An additional value was calculated per participant: the average duration of all their prospective estimations. This average (referred to as the “individual average of prospective estimates”) was used to examine possible correlations between individual factors such as age and musical sophistication and prospective estimates.

As all listeners responded both to the retrospective and the prospective questions, and the retrospective question was presented first, the comparison between the two paradigms (a person’s retrospective estimation, and their prospective estimate when the same stimulus was presented again) was not viable, as differences in duration judgements were likely to happen due to time-order errors (TOE). Considering this fact, retrospective and prospective judgements were analysed separately, and not compared.

### Results

Retrospective results will be described first, followed by prospective results. For each approach, we will start by analysing any relationship between tonality, tempo, and verbal estimates. Next, we will examine the possible correlations between estimates, attention, age, Gold-MSI subscales, and the ability to play an instrument.

#### Retrospective condition

To account for the eventuality that participants recruited via social media knew about this research and its intent, after providing the first duration estimate, participants were asked whether they knew, or suspected, that this experiment would be about time perception. Eight participants who answered positively were excluded from this analysis. Eight retrospective estimates were considered outliers and excluded. The sample size for the retrospective condition was thus 134.

There was a clear tendency to overestimate (*MD* = 1.59, *SD* = 0.75), with relative estimates ranging from 0.38 to 3.79 times chronometrical duration. One must note that the mean, in this case, will always lean towards higher values, as overestimates can be several times higher than the chronometrical duration, but underestimates are never equal or minor to 0. There were 36 underestimates, 97 overestimates, and 1 exact estimate.

##### Tonality and tempo

A two-way ANOVA using the retrospective relative estimates as the dependent variable and tonality and tempo as factors showed no significant main effect of tonality, *F*(1, 130) = 0.362, *p* = .549, η^2^ = .003, or tempo, *F*(1, 130) = 0.537, *p* *=* .465, η^2^ = .004, on duration judgements. There was also no significant interaction between tonality and tempo on estimates, *F*(1, 130) = 0.449, *p* = .504, η^2^ = .003.

##### Attention, Gold-MSI, age, and the ability to play an instrument

**Figure 2.**
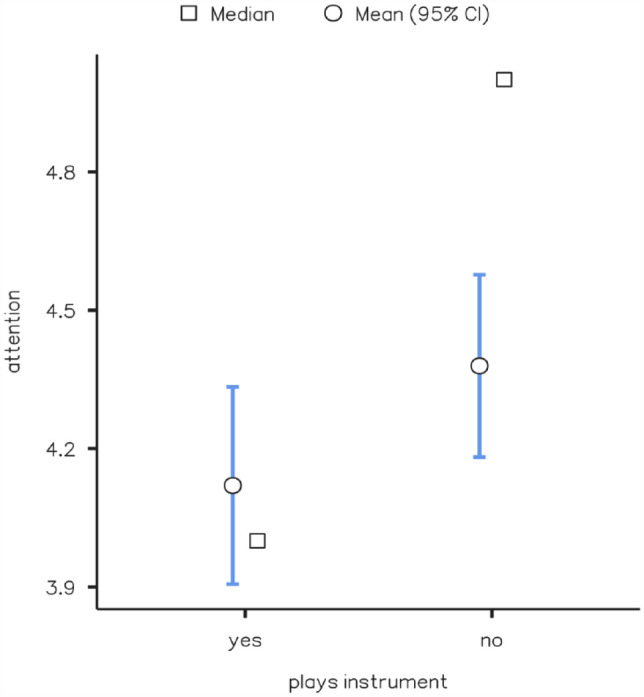
Self-assessments of attention in the retrospective condition, according to the ability to play an instrument.

Participants evaluated the level of attention they dedicated to the music as consistently high (*M* = 4.23, *SD* = 0.89), on a scale from 1 to 5. While the minimum and maximum values represent the full scale (min = 1.00, max = 5.00) the percentiles show little variability (25th = 4.00, 50th = 4.00, 75th = 5.00). A correlation matrix using the Pearson correlation coefficient and including retrospective estimates, age, attention, and Gold-MSI subscales revealed that estimates were not significantly affected by any of these variables. A Welch’s *t*-test comparing the retrospective estimates of participants who played one or more instruments (*n* = 75, *M* = 1.55, *SD* = 0.74) with those from participants who did not play an instrument (*n* = 59, *M* = 1.64, *SD* = 0.75) showed that this ability did not significantly affect retrospective estimates *t*(124) = −0.690, *p* = .492. A similar analysis comparing levels of attention of participants who played one or more instruments (*M* = 4.12, *SD* = 0.94) with those from participants who did not play an instrument (*M* = 4.38, *SD* = 0.77) revealed a marginally significant result *t*(131) = −1.746, *p* = .083 (Figure 2). However, a post hoc analysis calculated an achieved power of only 0.30, considering an effect size of 0.25 and an alpha of .05, meaning that this finding needs to be confirmed in future research before it can be considered as significant.

#### Prospective condition

As in the retrospective condition, there was a tendency to overestimate (*M* = 1.23, *SD* *=* 0.52), with average relative estimates ranging between 0.34 and 3.16. There was a total of 146 underestimates and 234 overestimates. Here *N* = sample × 4, as each participant provided one observation for each of all 4 possible musical combinations: 2 (tonal vs. atonal) × 2 (90 vs. 120 bpm).

##### Tonality and tempo

A two-way ANOVA revealed no significant main effect of tonality, *F*(1, 376) = 0.201, *p* = .654, η^2^ = .001, or tempo, *F*(1, 376) = 1.989, *p* = .159, η^2^ = .005, on estimates. There was also no significant interaction involving these musical factors, *F*(1, 376) = 0.002, *p* = .968, η^2^ = .000.

##### Attention, Gold-MSI, age, and playing an instrument

In the prospective condition participants evaluated their levels of attention as consistently high (*M* = 4.48, *SD* = 0.74) and with little variability, similarly to the retrospective condition. A correlation matrix using the Pearson correlation coefficient and including individual averages of prospective estimates, age, attention, and Gold-MSI subscales revealed that prospective estimates were not affected by any of these variables. When comparing individual averages of prospective estimates of participants who played one or more musical instruments (*n* = 58, *M* = 1.20, *SD* = 0.46) with participants who did not play any instrument (*n* = 37, *M* = 1.30, *SD* = 0.52), a Welch’s *t*-test showed no significant effect for this ability, *t*(70.4) = −0.919, *p* = .361. A post hoc analysis considering a moderate effect size (*f* = 0.25), and an alpha level of .05, calculated a power of only .320, so this result is not reliable enough to be considered.

### Discussion

This study intended to explore the influence of differences in tonality (tonal vs. atonal) and musical tempo on verbal estimates of duration, both retrospectively and prospectively. Additionally, musical sophistication was examined, and its possible link to estimates.

#### Tonality

The first hypothesis, that tonal music would be judged as longer retrospectively, and shorter prospectively, when compared with atonal music, was not supported by the results obtained. Given that a pilot study was conducted to verify that the musical stimuli used may indeed be readily perceived as tonal and atonal, respectively, a certain degree of confidence exists that the perception of tonality did vary between these two sets of extracts. One possible explanation for this first hypothesis not being supported by these results is that, although participants may have perceived differences in tonality, other features of the music may have taken priority in perception and overridden any significant duration estimation difference related to tonality. Besides hierarchical pitch structure, tonal and atonal music often differ in metric and rhythmic aspects (Mencke et al., 2019), and in pitch dispersal (Anta, 2017). Such typical musical features combinations were also present in the differences between tonal and atonal stimuli presented in Ziv and Omer’s (2010) experiment, which used music from the Baroque period and the 20th century. In our experiment, the rhythmic and metrical structures were unaltered between all conditions, which may have provided a strong organisation and structuring of the musical content and served as a guide for tracking and estimating time. Our variation between tonal and atonal stimuli was similar in process to the one assumed by Kellaris and Kent’s (1992) experiment, which produced different outcomes, perhaps due to other factors such as longer stimuli duration (2.5 min) or musical stimuli containing several musical lines and instruments.

#### Musical tempo

Our hypothesis concerning the effects of musical tempo on verbal estimates was also not supported by the results. This finding could perhaps be owing to the length of the stimuli, and future studies could explore the use of longer stimuli, as a potential acceleration of the internal pacemaker provoked by a faster tempo could be more noticeable with longer stimuli, given the scalar property of internal clock models. The musical tempos of 90 and 120 bpm were chosen owing to the tapping results of the pilot study, in order to select tempos that were in line with most people’s spontaneous tapping tempo, and in order for them to easily induce the same metrical level as the most salient pulse. It is possible that participants entrained to a greater variety of metrical levels than anticipated, and this factor impacted estimates to a greater extent than the speed of the extract. It could also be that the two chosen tempos were not sufficiently different from one another for a difference to be present. A previous study found that a faster tempo led to higher arousal (Droit-Volet et al., 2013). Maybe the use of faster tempos than the ones chosen for this experiment could lead to higher arousal levels, which could, in turn, cause the internal pacemaker to run faster. Further studies using longer musical stimuli, with more distant musical tempos, while keeping all remaining musical characteristics stable, could test these possibilities.

#### Musical sophistication

Musical sophistication did not impact verbal estimates, hence this hypothesis was not supported. Gold-MSI subscales scores were not significantly correlated with verbal estimates and did not interact with changes in tonality or tempo. This suggests that even if there were differences in the ways that more and less musically sophisticated participants perceived tonality and musical tempo, those differences did not impact their judgements about the music duration. Phillips and Cross (2011) found that musicians estimated the duration of a 37-s musical excerpt as shorter (less-overestimated) than non-musicians, in a retrospective experiment. Although this trend was also reflected in the results from this experiment it did not reach statistical significance. However, the musical stimulus in Phillips and Cross’ experiment was almost twice as long as most of our stimuli. Our results did show a marginally significant difference between the amount of attention paid to the music in the retrospective condition by participants who played an instrument or not. Perhaps, given the known interaction between attention and duration estimates, this difference in attention between musicians and non-musicians could be large enough to cause changes in verbal estimates with the use of longer musical stimuli, as the ones used by Phillips and Cross (2011). Still, considering the low variability in self-reported attention levels, and the low power achieved in this comparison, this relationship between attention paid and playing an instrument may not be reliable, and needs further exploration in future experiments. Given that this experiment used a reduced version of the Gold-MSI questionnaire it is possible that these questions, chosen automatically by the Gold-MSI configuration app, did not fully reflect aspects of musical expertise that would be important to affect perceptions of musical duration. It is possible that a higher emphasis on subscales such as musical training and perceived abilities, which relate more with Ericsson and Pool’s (2016) concept of expertise and the ability to create and manipulate mental representations of music, would be more relevant to express how differences in the processing of musical information may potentially impact the perception of duration during music listening.

#### Attention and duration estimates

Levels of attention, as self-reported by the participants, showed little variability, and did not correlate significantly with judgements of duration, as expected according to models of time perception. One may hypothesise that participants were not effective at evaluating the amount of attention dedicated to the music, since the amount of attention can be in itself a subjective concept, with different people having different focusing abilities. Also, people can only report the attention they were aware of, and another kind of non-conscious attention or awareness of the music could maybe impact time perception without participants noticing it. Another possibility is that participants may have simply taken their tasks seriously and paid attention to all musical stimuli equally, despite musical changes and potential differences in enjoyment. If we assume that participants did evaluate their attention levels reasonably accurately, then we may conclude that with musical stimuli within a range of 16–26 s, small differences in amounts of attention were not enough to cause a significant impact on time perception, and that the context of an experiment (even when conducted online) influenced participants to maintain a stable and high level of attention.

#### Unexplained variability and limitations

The descriptive statistics show a great amount of variability among the participants’ verbal estimates (relative estimates vary between 0.38 and 3.79 in the retrospective condition and between 0.34 and 3.16 in the prospective condition), which cannot be explained by the data gathered in this experiment. Some of this study’s limitations, which will now be described, may provide clues about potential factors related to this variability.

Verbal estimation may have been too unreliable a method to capture participants’ experiences of duration, maybe causing participants to provide round numbers, usually to the closest 10th (e.g., 30 s rather than 28), which happened in 37.0% of all estimates (retrospective and prospective combined). In fact, 24.2% of the participants who engaged in both prospective and retrospective conditions rounded more than half their estimates up or down to multiples of 10, even though the instructions asked them to be as precise as possible in their estimates and to avoid intentionally rounding values. This is a common tendency in verbal estimation already described in the literature as the process of “Quantization” (Block et al., 2018; Ogden et al., 2021; Wearden, 2016). The use of a reproduction method instead of a verbal estimation could maybe provide a closer insight into participants’ temporal experience, as it avoids the translation of a subjective experience into a numeric representation. Differences between these methods were reported by Hammerschmidt et al. (2021), who found that changes from slower to faster musical tempos were reflected in longer reproductions but not in longer verbal estimates.

The emotional state in which participants engaged in this experiment could potentially have affected time perception, a fact that was not accounted for. Wearden et al. (1999) observed that the participants’ emotional state impacts time perception and varies throughout the experiment. This possibility could not be analysed in this experiment, as the participants’ emotional state was not accounted for, and the order in which musical stimuli were randomised in the prospective condition was not recorded.

Participants were not asked about how much they enjoyed the music presented. Enjoyment has been linked to temporal estimates and perceived familiarity with music (Phillips & Cross, 2011; Ziv & Omer, 2010) and could perhaps explain some of the variability encountered.

Finally, although participants were instructed to use headphones, look for a calm environment, and avoid counting or looking at clocks, these aspects are impossible to control, given that the experiment was conducted online. As mentioned in the introduction, the context in which music listening takes place may influence how time is perceived. In this experiment, the advantages of a larger sample and better resource management in terms of time and financial costs were considered more valuable than the better control of environmental conditions provided by a lab setting.

To account for most of these limitations a second experiment was conducted, which tested the same hypotheses as Experiment 1, but had some differences in the procedure.

## Experiment 2

### Method

#### Participants

A total of 141 participants were recruited. All participants engaged in the retrospective condition, and 94 of them also engaged in the prospective condition. In line with the sample size determined in Experiment 1, the number of observations was higher than required by the power analysis, in both conditions. Participants were recruited online through social media and the platform Prolific Academic. Musical expertise was calculated using the Gold-MSI scoring app, as in Experiment 1. Participants’ age and musical expertise for retrospective and prospective conditions are described in Table 4.

**Table 4.** Descriptive statistics of age and musical expertise in retrospective and prospective conditions, in Experiment 2.

	Retrospective		Prospective	
	*M*	*SD*	*M*	*SD*
Age	32.12	9.46	34.5	9.53
Active engagement	4.18	1.33	4.17	1.28
Perceived abilities	4.93	1.28	5.04	1.21
Musical training	3.02	1.80	3.18	1.86
Singing abilities	4.16	1.26	4.33	1.27
Emotion	5.13	1.47	4.85	1.49
General sophistication	4.04	1.22	4.15	1.24

*SD*: standard deviation.

The study was granted ethical approval by the Centre for Interdisciplinary Research, based at the University of Coimbra, Portugal.

#### Materials

The materials used were the same as in Experiment 1.

#### Procedure

The procedure was similar to Experiment 1, with a few additions to address the limitations described above. First, before starting the experiment participants were asked to use fullscreen on their computers. This was an additional measure to prevent them from looking at the devices’ clocks, since for most devices, using the browser in fullscreen hides the clock display at the top or bottom of the screen. Second, participants were asked about their emotional state after providing their retrospective estimate. This questionnaire was the same as used by Wearden et al. (1999), which was a short version of Thayer’s activation–deactivation adjective checklist (Thayer, 1967). The self-report questionnaire asked participants to select from four options (Definitely feel, Feel slightly, Cannot decide, Definitely do not feel) the one which they felt most accurately applied to their present state, against each of the following adjectives: Energetic, Drowsy, Calm Jittery, Sleepy, Lively, Stirred-up and Relaxed. Third, after each duration estimate participants were asked how pleasurable the music they listened to was. They rated this from 1 to 5, with these numbers representing the following: very unpleasant, unpleasant, neutral, pleasant, and very pleasant. Fourth, duration estimates were made using the reproduction method, using a “start/stop” button, the method for reproducing time intervals that tends to lead to higher accuracy, according to Mioni et al. (2014). A fifth change made in Experiment 2 compared with Experiment 1 was that the order of the stimulis’ presentation in the prospective condition was recorded. Finally, choices of the questions in the Gold-MSI questionnaire were reviewed and edited to include more questions from the Musical Training and Perceived Abilities subscales.

#### Data analysis

Estimates were processed and analysed in the same way as in Experiment 1. Reproduction estimates under 1 s were considered unintentional double clicks of the “start/stop” button and therefore excluded. In the emotional state questionnaire answers were scored from 1 to 4 in the following order: Definitely do not feel, Cannot decide, Feel slightly, Definitely feel. Note that here scores were reversed in comparison with Wearden et al.’s (1999) procedure, with higher scores meaning more of the quality measured by the adjective. Scores from the four Activation adjectives (Energetic, Jittery, Lively, and Stirred-up) and the four Deactivation adjectives (Drowsy, Calm, Sleepy, and Relaxed) were categorised into two different groups. The Deactivation score was then subtracted from the Activation score. A final score <0 represented a deactivated state while a final score >0 corresponded to an activated state.

### Results

The description of results will follow the same order as in Experiment 1: retrospective results will be described first, followed by prospective results. For each paradigm, we will analyse possible relationships between reproduction estimates and tonality, tempo, attention, age, Gold-MSI subscales, and the ability to play an instrument.

#### Retrospective condition

Six retrospective estimates were excluded, as participants already knew, or suspected, that this experiment would be about time perception before taking part. Six additional retrospective estimates were considered outliers and excluded. The sample size for the retrospective condition was thus 129.

Contrary to Experiment 1, there was a clear tendency towards underestimation (*MD* = 0.93, *SD* *=* 0.29), with reproduction estimates ranging from 0.23 to 1.60 times the real duration. There were 78 underestimates and 51 overestimates.

##### Tonality and tempo

**Figure 3.**
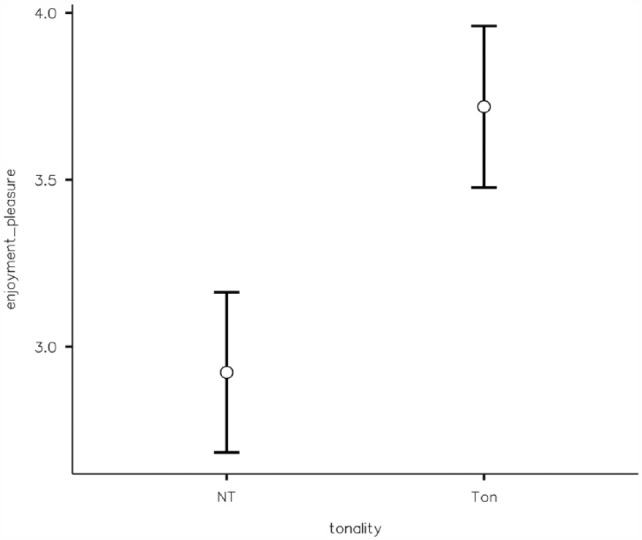
Effects of presence/absence of a tonal hierarchy on retrospective ratings of enjoyment. (NT = Non-Tonal, Atonal; Ton = Tonal).

A two-way ANOVA revealed no significant main effect of tonality, *F*(1, 125) = 5.06^−5, *p* = .994, η^2^ = .000, or tempo, *F*(1, 125) = 2.17, *p* *=* .143, η^2^ = .017, on reproduction estimates. There was also no significant interaction between tonality and tempo, *F*(1, 125) = 1.28, *p* = .261, η^2^ = .010. Tonality affected the pleasure induced by music, with tonal music being judged as significantly more pleasurable (*MD* = 3.72, *SD* *=* 0.85) than atonal music (*MD* = 2.92, *SD* *=* 1.09), as shown by a Student’s *t*-test *t*(127) = −4.62, *p* < .001 (Figure 3). A post hoc analysis performed considering this Student’s *t*-test indicated a power of only 0.29, considering an effect size of 0.25 and an alpha of .05, so this last finding may not be reliable enough and needs further confirmation in future studies.

##### Gold-MSI, instrument playing, enjoyment, and attention

Participants evaluated the level of attention they dedicated to the music as relatively high (*M* = 4.24, *SD* = 0.83), on a scale from 1 to 5, and with little variability (percentiles: 25th = 4.00, 50th = 4.00, 75th = 5.00), similarly to Experiment 1. A correlation matrix using the Pearson correlation coefficient including retrospective relative estimates, age, attention, and Gold-MSI subscales revealed that estimates were not significantly affected by any of these variables. The enjoyment induced by the music listened to was positively correlated with attention (*r* = .297, *p* < .001), Singing abilities (*r* = .231, *p* = .008), Active engagement (*r* = .197, *p* = .025), Perceived Abilities (*r* = .182, *p* = .039), and General sophistication (*r* = .186, *p* = .035) subscales. A Welch’s *t*-test comparing the retrospective estimates of participants who played one or more instruments (*n* = 84, *M* = 0.95, *SD* = 0.28) with those from participants who did not play an instrument (*n* = 45, *M* = 0.91, *SD* = 0.30) suggested that this ability did not significantly affect retrospective estimates *t*(83.3) = 0.730, *p* = .467. However, a post hoc analysis calculated an achieved power of only 0.27, considering an effect size of 0.25 and an alpha of .05, meaning that this finding needs to be confirmed in future research.

##### Age and emotional state

No effect of age on reproduction estimates was found. Contrary to what was expected, and assumed as a limitation for Experiment 1, the participants’ emotional state did not relate to any other factor and did not affect estimates of duration.

#### Prospective condition

Two prospective estimations were excluded due to being either too long (higher than 500 times real duration, considered either an error, or a participant’s temporary leave) or a reported error (one participant messaged mentioning unintentionally clicking before the intended time interval, and provided the specific stimulus’ order). Reproduction estimates varied from 0.23 to 1.68 and, as in the retrospective condition, there was a tendency to underestimate (*M* = 0.91, *SD* *=* 0.22). There was a total of 248 underestimates, and 128 overestimates.

##### Tonality and tempo

**Figure 4.**
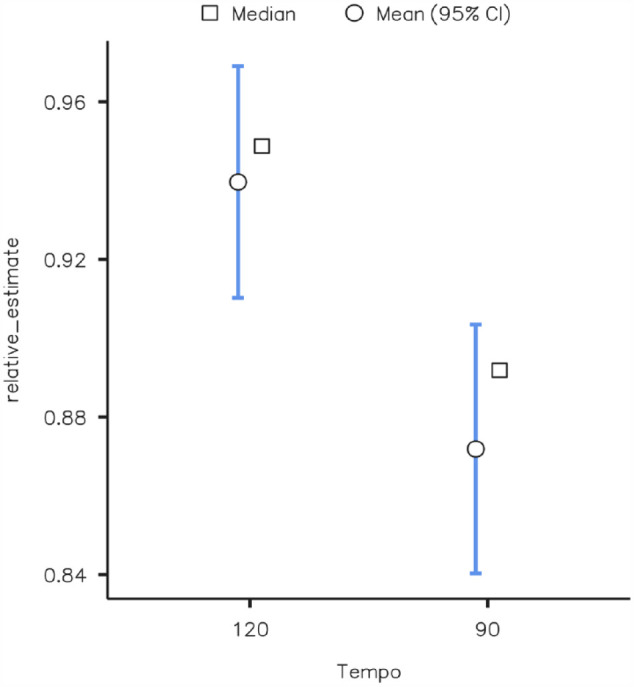
Effects of musical tempo on prospective relative estimates.

**Figure 5.**
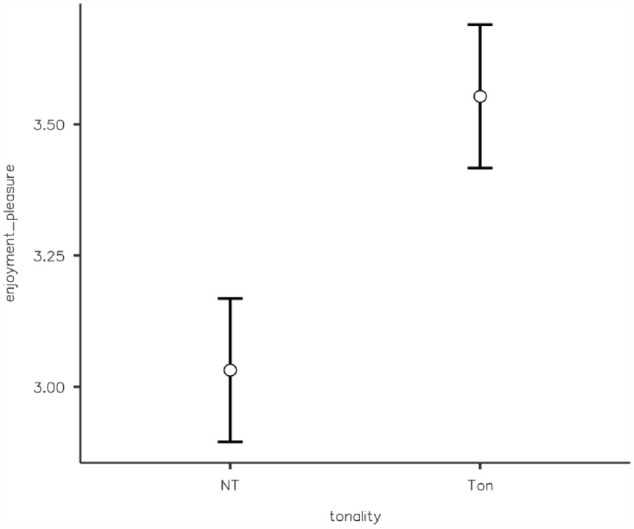
Effects of presence/absence of a tonal hierarchy on prospective ratings of enjoyment. (NT = Non-Tonal, Atonal; Ton = Tonal).

A two-way ANOVA revealed no significant main effect of tonality, *F*(1, 372) = 0.064, *p* = .801, η^2^ = .001, on reproduction estimates and no significant interaction between tonality and tempo *F*(1, 372) = 0.302, *p* = .583, η^2^ = .001. There was, however, a significant main effect of musical tempo, *F*(1, 372) = 9.432, *p* = .002, η^2^ = .025, on reproduction estimates, with a faster tempo associated with less underestimation (*M* = 0.94, *SD* = 0.21) than a slower tempo (*M* = 0.87, *SD* = 0.22) (Figure 4). Tonality affected the pleasure induced by music, with tonal music being judged as significantly more pleasurable (*M* = 3.55, *SD* = 0.92) than atonal music (*M* = 3.03, *SD* = 0.98), as shown by a Student’s *t*-test *t*(374) = −5.30, *p* < .001 (Figure 5).

##### Gold-MSI, instrument playing, enjoyment, and attention

In the prospective condition, participants also evaluated the level of attention they dedicated to the music as high (*M* = 4.33, *SD* = 0.87) and with low variability (25th = 4.00, 50th = 4.50, 75th = 5.00). A correlation matrix using the Pearson correlation coefficient and including individual averages of prospective estimates, age, attention, and Gold-MSI subscales revealed that prospective estimates were not affected by any of these variables. The enjoyment induced by the music listened to was positively correlated with Attention (*r* = 0.239, *p* = .020), Singing abilities (*r* = 0.234, *p* = .023), Active engagement (*r* = 0.258, *p* = .012), and General sophistication (*r* = 0.211, *p* = .041) subscales. Independent samples Welch’s *t*-tests showed no significant effect for the ability to play an instrument on estimates *t*(40.2) = −0.153, *p* = .879.

##### Age, emotional state, and order

Contrary to expectations, and what was assumed to be a limitation in Experiment 1, the participants’ emotional state did not impact prospective reproduction estimates and did not interact with other variables. The prospective relative estimates did not significantly correlate with the order in which stimuli were presented (*r* = −.054, *p* = .142), or age (*r* = −.181, *p* = .083).

Table 5 compares the results from both experiments, considering retrospective and prospective conditions, and the three factors addressed: tonality, musical tempo, and musical sophistication.

**Table 5.** Comparison of results from Experiments 1 and 2, according to conditions and initial hypotheses.

	Experiment 1 (verbal estimation)		Experiment 2 (reproduction)	
	Retrospective	Prospective	Retrospective	Prospective
Tonal plan (two-way ANOVA)	*F* = 0.362 η^2^ = .003	*F* = 0.201 η^2^ = .001	*F* = 5.06e-5 η^2^ = .000	*F* = 0.064 η^2^ = .000
Musical tempo (two-way ANOVA)	*F* = 0.537 η^2^ = .004	*F* = 1.989 η^2^ = .005	*F* = 2.170 η^2^ = .017	*F* = 9.432* η^2^ = .025
GMSI (Pearson correlation coefficient)				
Active engagement	*r* = −.112	*r* = −.128	*r* = −.039	*r* = −.125
Perceived abilities	*r* = −.005	*r* = −.104	*r* = .062	*r* = −.091
Musical training	*r* = −.016	*r* = −.055	*r* = .034	*r* = .166
Singing abilities	*r* = −.025	*r* = −.084	*r* = −.004	*r* = −.089
Emotion	*r* = −.113	*r* = −.052	*r* = .063	*r* = −.123
General sophistication	*r* = −.057	*r* = −.117	*r* = .037	*r* = −.037
Instrument playing (Welch’s *t*-test)	*t* = −0.690	*t* = −0.919	*t* = 0.730	*t* = −0.153

ANOVA: analysis of variance; GMSI: Goldsmiths Musical Sophistication Index.

* *p* < .05.

### Discussion

#### Tonality

Results from Experiment 2 suggest that changes in the presence of a pitch hierarchy did not affect the perception of duration, so our first hypothesis was not confirmed. This lack of evidence of an effect of tonality on duration estimates cannot be attributed to the chosen method, given that the reproduction method did demonstrate an influence of changes in musical tempo on time perception. Changes in tonality were not only clear enough to be seen in a probe test undertaken in the pilot study, but tonality also likely affected the amount of pleasure induced by the musical stimuli in participants. Although this last finding was not sufficiently powered in this experiment, and was not included in our initial hypothesis for tonality, these results are consistent with the existing literature. However, the amount of pleasure induced by the music did not influence reproduction estimates. One may still hypothesise, as in the discussion of Experiment 1 above, that to motivate changes in how duration is perceived the presence/absence of a pitch hierarchy has to be applied to longer stimuli and/or to be paired with differences in metrical and rhythmical organisation, as often is the case in most tonal and atonal music.

#### Musical tempo

Our second hypothesis, which posited that a faster musical tempo would lead to longer estimates in both prospective and retrospective paradigms, was partially confirmed.

Stimuli played at a faster tempo (120 bpm) were indeed judged as longer than the same stimuli played at a slower tempo (90 bpm), but this effect was only significant in the prospective condition. Results from the retrospective condition followed the same tendency but were far from significant levels (*p* = .119). This experiment shows that a relatively small difference in tempo may affect prospective estimates. Perhaps larger differences in tempo would lead to larger changes in prospective estimates, and/or some impact on retrospective estimates.

#### Musical sophistication

The degree of musical sophistication, as measured by a reduced version of the Gold-MSI, did not affect how participants judged the length of the musical stimuli, even when including more questions related to the concept of musical expertise proposed by Ericsson and Pool (2016), so our third hypothesis was not confirmed. The ability to play an instrument also did not affect duration judgements. As in Experiment 1, it is possible that even if there are differences in how more and less musically sophisticated participants perceive tonality and musical tempo, estimates of duration either rely on a level of perception of those variables that is common among listeners of different musical sophistication levels, or on other musical parameters that remained constant across stimuli, such as metric, rhythmic, or melodic structuring.

#### Emotional state, enjoyment, and order

Although these three factors were identified as possibly affecting estimates in Experiment 1, Experiment 2 suggested that this was not the case, at least when using the reproduction method. Although the level of enjoyment seems to be influenced by musical factors (tonality), individual factors (musical sophistication), and correlated with levels of attention, that did not directly impact reproduction estimates. Emotional state, which could impact a hypothetical internal clock used to estimate duration, did not affect reproduction estimates. One possible explanation is that music somehow levelled differences among emotional states reported at the beginning of the experiment, inducing a similar state among all participants. On the contrary, one may assume that the speed of the internal clock used to track the duration of a stimulus was the same used to reproduce its duration, so even if a participant with a more activated emotional state had a faster internal clock than a less activated participant, it may simply mean that the first participant would divide that time interval into a higher number of smaller time units. So, it is still possible that the participants’ emotional state affects prospective verbal estimates, but not reproductions of duration.

## General discussion

Tonality did not affect prospective estimates either in Experiment 1 or Experiment 2. Taken together, results from these experiments and similar studies suggest that the simple presence/absence of a pitch hierarchy (with no changes in other musical components such as rhythm, pitch dispersal, or metre) are not enough to influence duration estimates, even though these changes were noticeable enough to be perceived in a probe test as part of a pilot study. The level of the participants’ musical sophistication did not affect how they judged the duration of the musical stimuli (in both prospective and retrospective conditions), in either of the experiments. In line with some existing literature that suggests that a certain level of musical skill is acquired by the general population through simple exposure to music, one may hypothesise that these skills may be enough to level all individuals equally in terms of how music may affect their duration estimates, at least with the stimuli used in these experiments. In other words, music sophistication (including listening, playing, and other experience and knowledge) does not seem to impact estimates of duration during music listening.

Experiments 1 and 2 differed in the results regarding the influence of changes in musical tempo on prospective estimates, a finding similar to the one encountered by Hammerschmidt et al. (2021). Since the musical stimuli were the same in both experiments, it is possible that participants in Experiment 1 did experience different durations related to changes in musical tempo, but this difference was diluted when they translated that experience into numbers, given the fact that a considerable number of the estimates provided were rounded to the nearest 10. Expressing a temporal experience in numbers may also more easily have reflected differences in the emotional state among participants. In Experiment 1 participants with a more activated emotional state, and likely a more accelerated internal clock, may have counted more time units in a given interval, which would be associated with longer subjective judgements of duration. A comparison between Experiments 1 and 2 suggests that the reproduction method may be more suitable to investigate music’s direct effects on time perception than the verbal estimation method. Not only in Experiment 1 the verbal estimation method may have hidden the effects of changes in musical tempo on subjective duration (that were evidenced in Experiment 2) through the process of quantisation, but also it may be more prone to interferences from the listeners’ emotional state.

Another clear difference between Experiments 1 and 2 is the variability in under- and overestimations: while there was a tendency to overestimate in Experiment 1, the opposite happened in Experiment 2. This can be attributed to the differences in the methods used, as that was the main difference between both experiments. Such an explanation is in line with previous experiments (not focused on music listening, and using longer time periods) which have reported retrospective reproduction estimates as tending to be shorter than verbal estimates (Schiff & Thayer, 1968, 1970). Conversely, it is contrary to results from Hammerschmidt et al.’s (2021) experiment (using musical stimuli of similar duration to the ones used in Experiments 1 and 2), where prospective reproduction estimates tended to be longer than verbal estimates, and verbal estimates were consistently underestimated.

## Conclusion

To summarise, these two experiments investigated whether the presence/absence of a tonal hierarchy, changes in musical tempo, and level of musical sophistication (as measured by reduced versions of the Gold-MSI) affected listeners’ estimates of music duration. The presence of tonality did not significantly impact judgements of duration, suggesting that participants used other musical elements (such as the repetition of a regular rhythm, melodic contour, or metre across trials) to track time, or tonality may not impact the sense of time in the way that some previous studies have suggested. In the case of musical tempo, our hypothesis was partially supported, with a faster musical tempo leading to longer prospective estimates, but only when the reproduction method was used, and not with the verbal estimation method. This evidence suggests that the reproduction method, specifically using a “start/stop” button, may be more accurate than verbal estimation in expressing the differences felt by listeners’ temporal experience, at least when using relatively short stimuli (under half a minute). This represents a promising finding concerning methods for investigating the perception of duration in studies involving music. Although musical sophistication was related to the level of enjoyment experienced in music listening, it did not lead to differences in duration estimates. Age was also not linked to verbal or reproduction estimates of duration attributed to music. Experiment 2 accounted for extra factors, such as the enjoyment experienced in response to the musical stimuli, the order in which each stimulus was presented during the experiment, and the participants’ emotional state. This second study did not find any evidence that these variables were responsible for the differences in duration estimates encountered. Changes in musical tempo were not sufficient to fully explain those differences. Additional individual factors (such as personality traits) or contextual factors (such as environmental distractions, time of the day, or the sound quality of the devices used for listening) remain as variables that may account for those differences, which were not specifically examined in these experiments. This contextual issue highlights a potential disadvantage of conducting experiments in an online environment, where there is little control of the situation in which participants listen to the music (even despite remuneration and clear instructions being provided). In summary, these results build on existing research, which suggests that musical features and individual differences in musical sophistication may impact estimates of elapsed duration, and also demonstrate anew the complexities and fragilities of time perception while listening to music.

## Supplemental Material

sj-docx-1-qjp-10.1177_17470218231203459 – Supplemental material for The influence of tonality, tempo, and musical sophistication on the listener’s time-duration estimates Supplemental material, sj-docx-1-qjp-10.1177_17470218231203459 for The influence of tonality, tempo, and musical sophistication on the listener’s time-duration estimates by Ligia Borges Silva, Michelle Phillips and José Oliveira Martins in Quarterly Journal of Experimental Psychology
