# Supplementary material for: The influence of tonality, tempo, and musical sophistication on the listener’s time-duration estimates
Source: Q J Exp Psychol (Hove). 2023 Oct 31;77(9):1846–64. doi: 10.1177/17470218231203459 (PMC11373168; doi:10.1177/17470218231203459)

Supplementary Material for:

**The influence of tonality, tempo, and musical sophistication on the listener’s duration estimates**

Ligia Borges Silva^1^, Michelle Phillips^2^, José Oliveira Martins^1^ ^1^

^1^ Faculty of Arts and Humanities;

Centre for Interdisciplinary Studies (CEIS20);

Institute of Interdisciplinary Research;

University of Coimbra, Portugal.

^2^ Royal Northern College of Music, Manchester, United Kingdom.

**Authors note**

Lígia Borges Silva <https://orcid.org/0000-0001-5095-1929>

– Coimbra, Portugal - [ligia.silva@student.fl.uc.pt](mailto:ligia.silva@student.fl.uc.pt) and [ligiaborgessilva@hotmail.com](mailto:ligiaborgessilva@hotmail.com)

Michelle Phillips  <https://orcid.org/0000-0003-0933-3621> - Manchester, UK - [michelle.phillips@rncm.ac.uk](mailto:michelle.phillips@rncm.ac.uk)

José Oliveira Martins <https://orcid.org/0000-0002-7840-4705> – Coimbra, Portugal – [jmartins@uc.pt](mailto:jmartins@uc.pt)

**Musical scores:**

Melody A – Tonal version


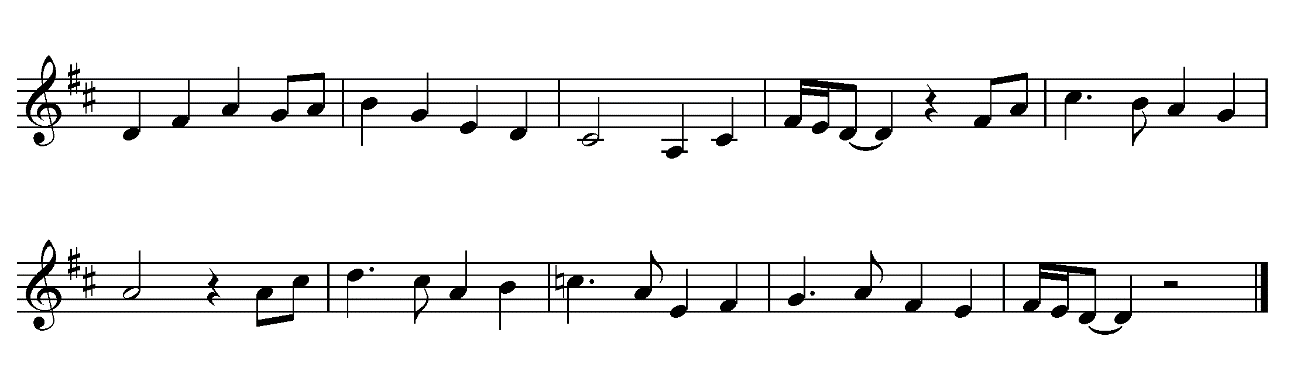


Melody A – Atonal version


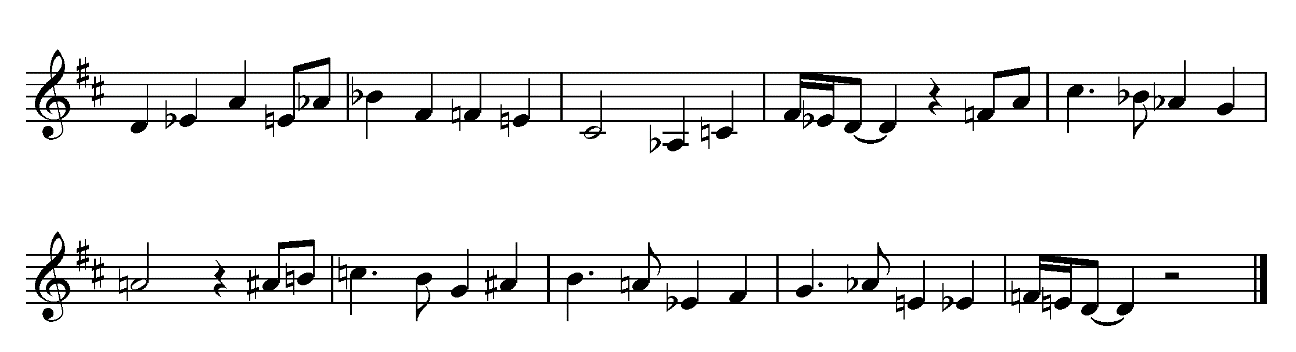


Melody B – Tonal version


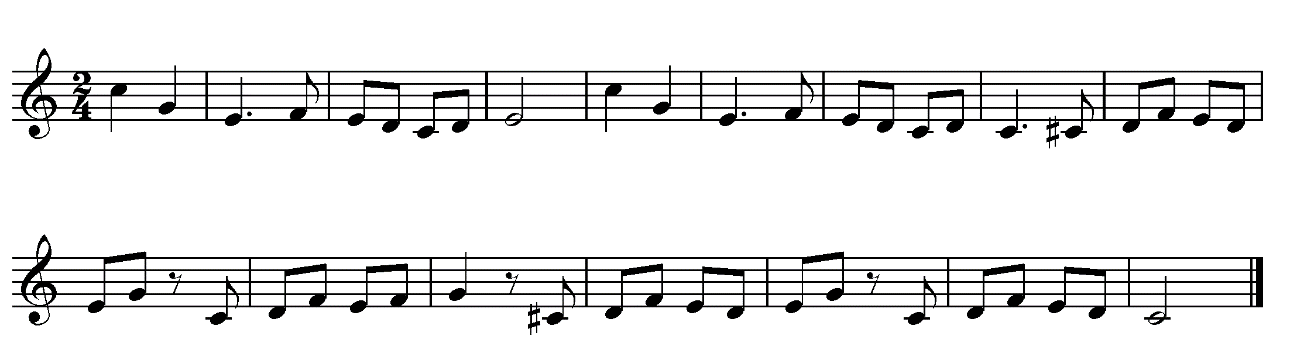


Melody B – Atonal version


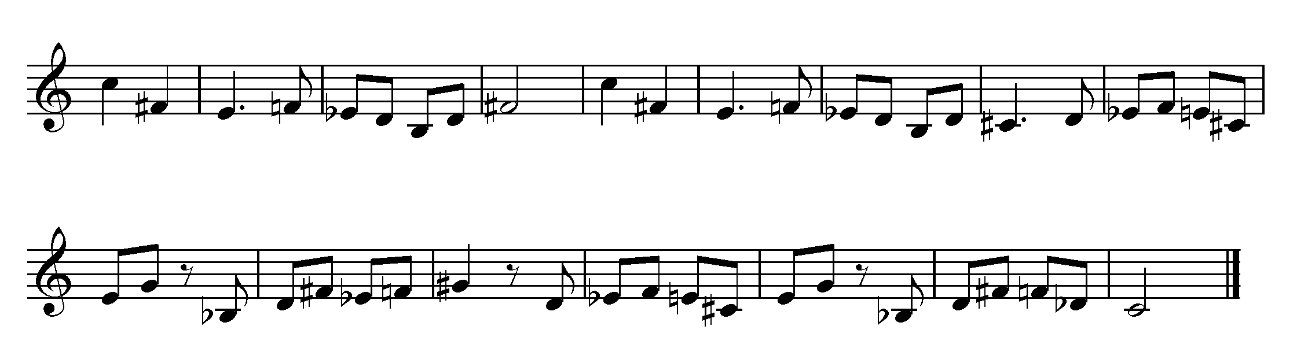

Supplement: sj-docx-1-qjp-10.1177_17470218231203459 – Supplemental material for The influence of tonality, tempo, and musical sophistication on the listener’s time-duration estimates [file sj-docx-1-qjp-10.1177_17470218231203459.docx]
